# Mundgesundheitstrends im Kindesalter: Ergebnisse aus den epidemiologischen Begleituntersuchungen zur Gruppenprophylaxe in Deutschland

**DOI:** 10.1007/s00103-021-03341-w

**Published:** 2021-06-07

**Authors:** Julian Schmoeckel, Ruth M. Santamaría, Roger Basner, Elisabeth Schankath, Christian H. Splieth

**Affiliations:** Zentrum für Zahn‑, Mund- und Kieferheilkunde, Abt. für Präventive Zahnmedizin & Kinderzahnheilkunde, Universitätsmedizin Greifswald, Walther-Rathenau-Straße 42, 17475 Greifswald, Deutschland

**Keywords:** Karies, Epidemiologie, Karieserfahrung, Mundgesundheit, Kinder, Caries, Epidemiology, Caries experience, Oral health, Children

## Abstract

Für die Beschreibung der aktuellen epidemiologischen Trends der Karieserfahrung bei Kindern in Deutschland wurden die Mundgesundheitsdaten primär anhand der *Epidemiologischen Begleituntersuchungen zur Gruppenprophylaxe* (DAJ-Studie) für das Schuljahr 2015/2016 dargestellt. Hier standen Kariesdaten von 301.684 Kindern verteilt auf 3 verschiedene Altersgruppen zur Verfügung. Für die Trends in der Kariesentwicklung wurden zudem weitere in Deutschland durchgeführte nationale und regionale Studien herangezogen.

Karies an Milchzähnen tritt schon sehr früh auf (bereits 10–17 % der 3‑Jährigen sind betroffen) und ist auch unter 6‑ bis 7‑Jährigen noch weitverbreitet (ca. 40–60 %). Sehr hoch ist dabei der Anteil nicht sanierter kariöser Milchzähne (3-Jährige: 73,9 %, 6‑ bis 7‑Jährige: 42,5 %). Bei den 6‑ bis 7‑Jährigen sind in den letzten 10 Jahren außerdem kaum noch Verbesserungen zu verzeichnen. Die Karieswerte der erstmals im Rahmen der DAJ-Studie national untersuchten 3‑Jährigen liegen in dem Bereich der Werte aus bisherigen lokalen Studien der letzten Jahrzehnte, bei den 6‑ bis 7‑Jährigen konvergieren die Werte regional. Bei den 12-Jährigen ist die mittlere Karieserfahrung im bleibenden Gebiss in den letzten knapp 20 Jahren um etwa 80 % gesunken. Damit liegt Deutschland hinsichtlich der Mundgesundheit dieser Altersgruppe weltweit im Spitzenbereich.

Bezüglich der Mundgesundheit im Milchgebiss besteht in Deutschland dennoch ein deutliches Optimierungspotenzial. Kürzlich implementierte präventive Maßnahmen adressieren dieses Problem bereits, sodass weitere Verbesserungen ähnlich den Erfolgen im bleibenden Gebiss realistisch erscheinen und zu erwarten sind.

## Abstract (en)

To describe the current epidemiological trends in the dental caries experiences of children in Germany, oral health data was primarily presented on the basis of the *Epidemiological Studies Accompanying Group Prophylaxis* (DAJ study) for the 2015/16 schoolyear. Caries data of 301,684 children from three different age groups were available. The trends in caries development were based on previous national and regional studies conducted in Germany.

Caries in the primary dentition occur very early (approx. 10–17% of 3‑year-olds) and are still widespread among 6‑ to 7‑year-olds (approx. 40–60%). The proportion of unrestored decayed primary teeth is very high (3-year-olds: 73.9%; 6 to 7‑year-olds: 42.5%). There has also been no significant improvement among 6‑ to 7‑year-olds in the last 10 years. The values for the 3‑year-olds that were nationally examined for the first time in the DAJ study are in the range of previous regional studies from recent decades. Caries prevalence among the 6‑ to 7‑year-olds shows a regional convergence. Among the 12-year-olds, mean caries experience in the permanent dentition has fallen by about 80% in the last 20 years. Regarding oral health in this age group, Germany ranks highest among countries worldwide.

There is still a substantial need for oral health improvement in the primary dentition of children in Germany. Recently implemented preventive measures already focus on tackling this problem. Further improvements in caries prevention similar to those in the permanent dentition are feasible and expected.

## Hintergrund und Ziel des Beitrags

Erfolge bei der Kariesprophylaxe lassen sich nur durch repräsentative, zahnmedizinische Vergleichsuntersuchungen beschreiben. Daher initiierte die Deutsche Arbeitsgemeinschaft für Jugendzahnpflege (DAJ) im Jahr 1993 die *Epidemiologischen Begleituntersuchungen zur Gruppenprophylaxe*, die in Schlüsselgruppen wie 6‑ bis 7‑, 9‑, 12- oder 15-Jährige in möglichst allen Bundesländern repräsentativ durchgeführt werden sollten [3]. Die *Epidemiologischen Begleituntersuchungen zur Gruppenprophylaxe* erfolgten daraufhin bisher in den Jahren 1994/1995, 1997, 2000, 2004, 2009 [1] sowie 2016 [2]. Sie erfassen in den einzelnen Bundesländern und auch auf nationaler Ebene die mittlere Karieserfahrung sowie die Kariesprävalenz in den verschiedenen Altersgruppen. Im Zuge des Kariesrückganges deutet sich eine im Wesentlichen sozial polarisierte Verteilung an. Deshalb wurde mit dem Significant Caries Index (SiC; [4]) ein Instrumentarium geschaffen, um das Drittel der Kinder mit dem höchsten Kariesbefall getrennt zu verfolgen und ihre Werte mit dem Mittelwert aller Kinder und Jugendlichen vergleichen zu können. Da aufgrund der Präventionserfolge jedoch die Kariesprävalenz in einigen Altersgruppen aktuell bereits unter 33 % liegt, wurde mit dem Specific Affected Caries Index (SaC; [5]) erst kürzlich zusätzlich ein weiterer Index eingeführt, um die Karieserfahrung in diesen Risikogruppen noch besser darzustellen.

Ziel dieses Übersichtsbeitrags ist die Beschreibung der aktuellen Karieserfahrung bei Kindern in Deutschland anhand nationaler Studien wie den *Epidemiologischen Begleituntersuchungen zur Gruppenprophylaxe* (DAJ-Studie 2016) für 3 Referenzaltersgruppen (3-, 6‑ bis 7‑ und 12-Jährige).

## Datengrundlage

Für diesen Übersichtsbeitrag wurden die aktuelle Mundgesundheit anhand der *Epidemiologischen Begleituntersuchungen zur Gruppenprophylaxe* (DAJ-Studie; [1, 2]) sowie die Kariestrends anhand verschiedener weiterer in Deutschland durchgeführter nationaler und regionaler Studien zur Mundgesundheit bei Kindern (u. a. in Sachsen-Anhalt, Brandenburg) dargestellt [2]. Dazu gehören für die 12-Jährigen auch die *Deutschen Mundgesundheitsstudien *des Instituts der Deutschen Zahnärzte (IDZ; [6]) und weitere repräsentative Studien zur Kariesepidemiologie aus den 1970er- bzw. 1980er-Jahren [7, 8]. So konnten für die Referenzgruppe laut Weltgesundheitsorganisation (WHO) von Jugendlichen (i. d. R. 12-Jährige; [9]) über 40 Jahre hinweg sowie für 6‑ bis 7‑jährige Grundschüler über knapp 20 Jahre hinweg die Entwicklungen in der Kariesprävalenz dargestellt werden. Erstmalig wurden in der aktuellen DAJ-Studie (Schuljahr 2015/2016) auch 95.127 3‑Jährige in Kindertagesstätten erfasst [2]. Die Befunderhebung in den Studien umfasst primär die Karieserfahrung anhand der WHO-Kriterien [10], d. h. für permanente Zähne den DMFT-Index bzw. bei Milchzähnen der dmft-Index („decayed“/kariöse, „missing“/extrahierte, „filled“/gefüllte „teeth“/Zähne), die erfassten Einzelkomponenten des Index sowie erstmalig 2016 auch die Initialkaries (i bzw. I). Die Kariesprävalenz bezeichnet den prozentualen Anteil der Kinder mit DMFT/dmft > 0. Hauptunterschiede zwischen der DAJ-Studie und der IDZ-Studie waren, dass bei der DAJ-Studie die Untersuchung als Begleituntersuchung zur Gruppenprophylaxe erfolgte, also unter Feldbedingungen in Kindertagesstätten und Schulen [2]. Bei der *Fünften Deutschen Mundgesundheitsstudie* (DMS V) des IDZ konnte auf einem Zahnarztstuhl, also unter praxisähnlicheren Bedingungen, untersucht werden [6]. Für die DAJ-Studie 2016 wurden die Kariesdaten von 301.684 Kindern durch 482 kalibrierte zahnärztliche Untersucher (Onlinekalibrierung) verteilt auf 3 verschiedene Altersgruppen herangezogen [2], während in der DMS V eine national repräsentative Stichprobe gezogen wurde und lediglich 1468 12-Jährige (Geburtsjahrgang 2002) untersucht wurden [6].

## Ergebnisse

### 3-Jährige in Kindertagesstätten

3‑Jährige in Kindertagesstätten (*n* =95.127) wurden erstmalig im Rahmen der *Epidemiologischen Begleituntersuchungen* untersucht und wiesen eine mittlere Karieserfahrung von 0,5 dmft auf. Der Anteil der Kinder, der in diesem Alter bereits eine manifeste Karies aufweist, schwankt je nach Bundesland zwischen 10,5 % und 16,2 % (Tab. 1), was sich unter Einbeziehung von Initialläsionen auf 14,4–24,6 % Kinder mit Karieserfahrung erhöht. Dies entspricht der Prävalenz von frühkindlicher Karies (ECC: Early Childhood Caries) aus zahlreichen regionalen Studien [11] und den in der Selbstbeschreibung der einzelnen Bundesländer angegebenen Daten zur Karieserfahrung bei 3‑Jährigen [2]. Das Ausmaß der Polarisation des Kariesbefalls wird insbesondere bei Betrachtung des SiC und SaC über alle Bundesländer hinweg deutlich (Tab. 1), da vergleichsweise wenige Individuen eine hohe Karieslast auf sich vereinen (11,4 %), während die große Mehrheit in diesem Alter (noch) gesunde Zähne aufweist. So lag die mittlere Karieserfahrung der Kinder mit Karieserfahrung (SaC) bereits bei 3,6 dmft. Zudem waren etwa Dreiviertel der kariösen Milchzähne bei den 3‑Jährigen nicht saniert [2] und folglich sowohl der Präventions- als auch der Therapiebedarf hoch. Klinisch tritt die ECC meist zunächst als sogenannte Nuckelflaschenkaries an den Oberkieferschneidezähnen auf, was auch erklärt, warum die von Karies betroffenen Kinder im Schnitt fast 4 kariöse Zähne haben.

**Table 1:** 

Bundesland/Region	*N*	dmft	it	dt	mt	ft	SiC	SaC	Sanierungsgrad (%)	Kinder mit dt (%)	dmft = 0 (%)
Baden-Württemberg	Keine Untersuchung von 3‑Jährigen										
Bayern	Keine Untersuchung von 3‑Jährigen										
Berlin	16.453	0,58	0,29	0,41	0,05	0,13	1,75	3,61	30,0	13,3	83,8
Brandenburg	14.337	0,48	0,17	0,37	0,03	0,08	1,43	3,55	23,3	11,4	86,5
Bremen	Keine Untersuchung von 3‑Jährigen										
Hamburg	Keine Untersuchung von 3‑Jährigen										
Hessen	Keine Untersuchung von 3‑Jährigen										
Mecklenburg-Vorpommern	8241	0,51	0,18	0,41	0,03	0,07	1,52	3,5	20,1	12,3	85,5
Niedersachsen	4870	0,52	0,14	0,41	0,04	0,07	1,56	3,76	21,5	12,0	86,2
Nordrhein	1849	0,39	0,18	0,26	0,05	0,08	1,17	3,48	35,0	9,3	88,8
Rheinland-Pfalz	Keine Untersuchung von 3‑Jährigen										
Saarland	Keine Untersuchung von 3‑Jährigen										
Sachsen	22.479	0,40	0,16	0,31	0,03	0,07	1,21	3,47	23,4	10,0	88,4
Sachsen-Anhalt	9415	0,58	0,18	0,43	0,05	0,10	1,73	3,48	25,2	13,8	83,4
Schleswig-Holstein	5530	0,38	0,16	0,26	0,04	0,07	1,15	3,65	30,9	8,2	89,5
Thüringen	10.523	0,56	0,25	0,42	0,04	0,10	1,67	3,5	24,6	13,3	84,1
Westfalen-Lippe	1430	0,49	0,2	0,35	0,06	0,07	1,48	3,72	26,8	10,6	86,8
Deutschland	95.127	0,48^a^	0,19	0,36	0,04	0,08	1,47	3,57	26,1	11,4	86,3

*dmft* „decayed, missing, filled primary teeth“: Index für die Karieserfahrung im Milchgebiss

*it* initialkariöser Milchzahn, *dt* defektkariöser Milchzahn, *mt* extrahierter Milchzahn, *ft* gefüllter Milchzahn

*Sanierungsgrad: *Anteil sanierter Milchzähne am dmft

*dmft* *=* *0:* Anteil gesunder Gebisse in %

*Kinder mit dt:* prozentualer Anteil der Kinder mit unbehandelten defektkariösen Zähnen

*SaC*_*dmft*_, Specific Affected Caries Index: mittlerer dmft in der Gruppe mit dmft > 0 [5]

*SiC*_*dmft*_, Significant Caries Index: mittlerer dmft-Wert des einen Drittels der Kinder mit den höchsten Karieswerten [4]

^a^ dmft-Wert für Deutschland nach Bevölkerung gewichtet

Trotz Einschränkungen bei der Gewinnung von 3‑Jährigen in Kinderkrippen und Kindergärten zur Studienteilnahme lässt sich durch zahnärztliche Untersuchungen schon hier eine Polarisation des Kariesbefalls auf eine Gruppe von ca. 15 % der Kinder bzw. eine Hochkariesrisikogruppe (dmft > 4) von etwa 5 % mit vorwiegend unbehandelten kariösen Defekten identifizieren.

Im Rahmen der DAJ-Studie sind keine nationalen Vergleichsdaten für die Altersgruppe der 3‑Jährigen aus früheren Untersuchungen verfügbar. Jedoch konnte für Mecklenburg-Vorpommern auf Vergleichsdaten aus der Gesundheitsberichterstattung der letzten 20 Jahre zurückgegriffen werden, die einen Kariesrückgang von 1,0 dmft (1992) um 50 % auf aktuell 0,5 dmft erkennen lassen [12]. Auch in Brandenburg lässt sich anhand der online verfügbaren Daten vom Landesamt für Arbeitsschutz, Verbraucherschutz und Gesundheit in den letzten 10 Jahren eine leichte Verbesserung der Mundgesundheit verzeichnen: So stieg der Anteil naturgesunder Gebisse bei 3‑Jährigen von 83,4 % (2010) auf 87,2 % (2019) und zugleich sank in diesem Zeitraum die mittlere Karieserfahrung von 0,59 auf 0,52 dmft [13].

### 6- bis 7-Jährige in der 1. Klasse

Insgesamt wurden in der aktuellen DAJ-Studie [2] 151.555 6‑ bis 7‑Jährige in der 1. Klasse untersucht. Die Kariesprävalenz im Milchgebiss sank von 65 % im Jahr 1994 auf 44 % im Jahr 2016 (Tab. 2), während die mittlere Karieserfahrung von 2,89 auf 1,73 dmft (dt = 0,74, mt = 0,19, ft = 0,80) sank (Tab. 3). Der Sanierungsgrad der kariösen Zähne betrug 57,5 %, und der SiC 4,84 dmft. Je nach Region variierte der mittlere dmft stark und lag zwischen 1,37 und 2,31 (Tab. 3). Zusammenfassend lässt sich sagen, dass trotz des allgemeinen Kariesrückgangs bei den 6‑ bis 7‑Jährigen in Deutschland in den letzten 10 Jahren nur geringe Kariesreduzierungen beobachtet wurden, wobei ein immer noch hoher Anteil an unbehandelter Karies besteht [2]. Dies tritt klinisch mitunter als multiple approximale kariöse Läsionen an Milchmolaren in Erscheinung und stellt bei einer Kariesprävalenz von knapp jedem zweiten Kind in der 1. Klasse somit kein ausschließlich soziales Randphänomen dar. Effektivere präventive und restaurative Maßnahmen mit Schwerpunkt Milchgebiss sind daher nötig [14].

**Table 2:** 

Bundesland/Region	Mittlere Karieserfahrung bei 6‑ bis 7‑Jährigen der 1. Klasse (dmft)						Kariesreduktion (%)
Jahr	1994/1995	1997	2000	2004	2009	2016	Bis 2016
Baden-Württemberg	2,40	1,91	1,72	1,58	–	1,85	22,9
Bayern	–	–	–	2,35	2,36	1,37	41,7^a^
Berlin	3,10	2,64	2,33	2,74	2,40	2,13	31,3
Brandenburg	–	2,54	2,43	2,76	2,20	1,85	27,2
Bremen	3,10	2,68	3,27	2,76	2,40	1,92	38,1
Hamburg	2,70	2,20	2,24	1,84	1,68	1,70	37,0
Hessen	2,80	2,30	1,98	2,06	1,75	1,81	35,4
Mecklenburg-Vorpommern	4,00	3,04	2,95	2,58	2,26	2,23	44,3
Niedersachsen	2,90	2,59	2,36	2,09	1,78	1,78	38,6
Nordrhein	3,00	2,90	2,30	2,05	1,75	1,59	47,0
Rheinland-Pfalz	–	–	2,14	2,01	1,78	1,53	28,5^b^
Saarland	–	–	–	2,00	1,30	1,51	24,5^a^
Sachsen	–	–	–	2,33	1,89	1,75	24,9^a^
Sachsen-Anhalt	3,82	3,20	3,06	2,91	2,31	2,31	39,5
Schleswig-Holstein	2,50	1,90	1,60	1,69	1,45	1,47	41,2
Thüringen	3,75	2,92	2,41	2,78	2,56	2,08	44,5
Westfalen-Lippe	2,80	2,22	2,27	2,27	1,90	1,78	36,4
*Deutschland (insgesamt)*							
dmft	2,89	2,39	2,21	2,16	1,87	1,73	40,1
Kariesprävalenz	65 %	57 %	53 %	52 %	48 %	44 %	21 %

Werte für Deutschland gewichtet nach Bevölkerung auf Basis der Ergebnisse in den Epidemiologischen Begleituntersuchungen zur Gruppenprophylaxe von 1994 bis 2016 (Daten für 1994–2009 übernommen von [1], Daten 2016 [2])

*dmft* „decayed, missing and filled teeth“: Index zur Karieserfahrung im Milchgebiss

^a^Kariesreduktion berechnet seit 2004

^b^Kariesreduktion berechnet seit 2000

**Table 3:** 

Bundesland/Region	*n*	dmft	it	dt	mt	ft	SiC	SaC	Sanierungsgrad in %	Kinder mit dt in %	dmft = 0 in %
Baden-Württemberg	2310	1,85	1,31	0,81	0,18	0,86	5,11	4,01	56,4	29,4	53,8
Bayern	2874	1,37	0,24	0,51	0,10	0,75	3,94	3,52	62,8	20,4	61,2
Berlin	21.934	2,13	0,34	0,78	0,29	1,05	5,67	4,20	63,2	30,2	49,4
Brandenburg	17.227	1,85	0,19	0,81	0,16	0,88	5,03	3,89	56,1	29,7	52,4
Bremen	1888	1,92	0,41	0,97	0,17	0,78	5,11	3,85	49,3	34,5	50,2
Hamburg	3458	1,70	0,49	0,62	0,22	0,86	4,86	4,10	63,4	22,9	58,4
Hessen	2324	1,81	0,26	0,61	0,24	0,96	5,10	4,17	66,3	23,9	56,5
Mecklenburg-Vorpommern	2643	2,23	0,30	0,96	0,25	1,02	5,57	3,87	57,0	36,3	42,6
Niedersachsen	2281	1,78	0,28	0,77	0,18	0,83	5,00	4,11	56,7	28,3	56,8
Nordrhein	5268	1,59	0,21	0,70	0,22	0,67	4,60	4,11	56,2	24,5	61,4
Rheinland-Pfalz	2610	1,53	0,15	0,95	0,17	0,42	4,43	3,95	38,2	29,0	61,2
Saarland	1941	1,53	0,08	0,72	0,28	0,53	4,37	3,74	53,2	25,5	59,1
Sachsen	28.989	1,75	0,25	0,74	0,18	0,84	4,84	3,82	58,0	27,9	54,1
Sachsen-Anhalt	13.965	2,31	0,19	1,08	0,27	0,96	5,88	4,16	53,3	36,0	44,4
Schleswig-Holstein	24.083	1,47	0,19	0,61	0,18	0,69	4,26	3,82	58,8	22,2	61,5
Thüringen	14.566	2,08	0,22	0,89	0,22	0,97	5,45	3,98	57,2	31,9	47,7
Westfalen-Lippe	3194	1,78	0,20	0,85	0,22	0,71	5,02	4,09	52,0	29,5	56,4
Deutschland^a^	*151.555*	1,73	0,38	0,74	0,19	0,80	4,84	3,97	57,5	26,9	56,4

*dmft* „decayed, missing, filled primary teeth“: Index für die Karieserfahrung im Milchgebiss

*it* initialkariöser Milchzahn, *dt* defektkariöser Milchzahn, *mt* extrahierter Milchzahn, *ft g*efüllter Milchzahn

*Sanierungsgrad*: Anteil sanierter Milchzähne am dmft

*dmft* *=* *0:* Anteil gesunder Milchgebisse in %

*Kinder mit dt*: prozentualer Anteil der Kinder mit unbehandelten defektkariösen Zähnen

*SaC*_*dmft*_*,* Specific Affected Caries Index: mittlerer dmft in der Gruppe mit dmft > 0 [5]

*SiC*_*dmft*_*,* Significant Caries Index: mittlerer dmft-Wert des einen Drittels der Kinder mit den höchsten Karieswerten [4]

^a^dmft-Wert für Deutschland nach Bevölkerung gewichtet

### Jugendliche (12-Jährige)

Im zeitlichen Verlauf sind insbesondere in dieser Altersgruppe deutliche Verbesserungen der Mundgesundheit zu beobachten. In den letzten knapp 20 Jahren, in denen die DAJ-Studie durchgeführt wurde, konnte der mittlere DMFT von 2,44 um ungefähr 80 % reduziert werden (Tab. 4). So wiesen 12-Jährige der Klassenstufe 6 in der aktuellen DAJ-Studie 2015/2016 [2] eine mittlere Karieserfahrung von 0,44 DMFT auf, wobei 79 % der Kinder keine kavitierten kariösen Läsionen hatten (DMFT = 0, Tab. 5). Diese Ergebnisse liegen sehr nahe den ermittelten Werten der DMS V (Untersuchungsjahr 2014), in der die 12-Jährigen einen DMFT von 0,5 und eine Kariesprävalenz von 19 % aufwiesen [6]. Dies verdeutlicht trotz unterschiedlicher Methodik und potenziellen Selektionsbias in beiden Studien die Robustheit der Kariesmarker (insbes. des DMFT). Zudem konnte anhand zusätzlicher Daten aus Sachsen-Anhalt, wo im Rahmen der Gesundheitsberichterstattung 12-Jährige unabhängig von der Klassenstufe 6 untersucht wurden, ein einfacher Adjustierungsfaktor (DMFT + 15 %) zur Kompensation des Selektionsbias im Rahmen der DAJ-Studie ermittelt werden (s. DAJ-Gutachten, Kap. 7.3.3 [2]).

**Table 4:** 

Epidemiologische Studie, Jahr	DMFT	DT	MT	FT	IT	SiC_DMFT_	Prävalenz DMFT = 0, %	SaC_DMFT_
ICS 1 Hannover/Region, 1973^a^	8,8	4,4	0,4	4,1	–	–	1	8,9^g^
ICS 1 Leipzig/Region, 1979^a^	4,9	1,6	0,2	3,0	–	–	10	5,4^g^
A5 Westdeutschland, 1983^b^	8,8	2,8	0,3^e^	5,7	–	–	2	9,0^g^
IDZ Westdeutschland, 1989^c^	5,1	2,1	0,1	3,0	–	9,6^f^	13	5,9^g^
A10 Westdeutschland, 1989^b^	6,4	3,2	0,2	–	–	–	11	7,2^g^
IDZ Ostdeutschland, 1992^c^	4,3	0,7	0,1	3,5	–	8,4^f^	16	5,1^g^
DAJ, 1994/1995^d^	2,44	0,43	0,05	1,98	–	5,33	30	3,5^g^
DAJ, 1997^d^	1,75	0,33	0,03	1,44	–	4,46	39	2,8^g^
IDZ, 1997^d^	1,7	0,4	0,0	1,3	3,0	4,1	42	2,9^g^
DAJ, 2000^d^	1,21	0,23	0,03	0,98	–	3,43	52	2,5^g^
DAJ, 2004^d^	0,98	0,24	0,03	0,68	–	2,99	60	2,5^g^
IDZ, 2004^d^	0,7	0,7	0,2	0,5	0,9	2,1	70	2,3^g^
DAJ, 2009^d^	0,72	0,22	0,02	0,48	–	2,18	69	2,3^g^
IDZ, 2014^d^	0,5	0,1	0,1	0,3	0,6	1,4	81	2,6^g^
DAJ, 2016^d^	0,44	0,14	0,02	0,29	0,52	1,33	79	2,1

Erste Umfragen der Weltgesundheitsorganisation (WHO) bei 13- bis 14-Jährigen für Hannover/FRG und Leipzig/DDR [8], Umfragen in der Privatpraxis in ganz Deutschland (A5-A10-Studien [7]), bevölkerungsregisterbasierte Umfragen des IDZ [6] und schulbasierte nationale Mundgesundheitsuntersuchung bei 12-Jährigen in der 6. Klasse durch die DAJ [2]. Adaptiert nach Splieth et al. [15]

*DMFT* „decayed, missing, filled permanent teeth“: Index für die Karieserfahrung im bleibenden Gebiss

*IT* initialkariöser Zahn, *DT* defektkariöser Zahn, *MT* extrahierter Zahn, *FT* gefüllter Zahn

*Sanierungsgrad:* Anteil sanierter Zähne am DMFT

*DMFT* *=* *0:* Anteil gesunder Gebisse in %

*Kinder mit DT:* prozentualer Anteil der Kinder mit unbehandelten defektkariösen Zähnen

*SaC*_*DMFT*_ Specific Affected Caries Index: mittlerer DMFT in der Gruppe mit DMFT > 0 [5]

*SiC*_*DMFT*_ Significant Caries Index: mittlerer DMFT-Wert des einen Drittels der Kinder mit den höchsten Karieswerten [4]

^a^WHO, 13- bis 14-Jährige, Mittelwert zwischen städtischem und nichtmetropolitanem Raum [8]

^b^A‑Studien, 13- bis 14-Jährige in Zahnarztpraxen [7]

^c^IDZ (Institut Deutscher Zahnärzte), 13- bis 14-Jährige, repräsentative Stichprobe [16, 17]

^d^DAJ (Deutsche Arbeitsgemeinschaft für Jugendzahnpflege) und IDZ-Studien, repräsentative Stichprobe von 12-Jährigen, DAJ erhoben in der 6. Klasse [2, 6]

^e^Der ursprüngliche Wert betrug 2,3 MT unter Einbeziehung kieferorthopädisch bedingter Prämolarenextraktionen, die von der Studie später auf 0,3 MT umgerechnet wurden

^f^Berechnet aus der Verteilung der Originaldaten

^g^Berechnet aus den Originaldaten: $100\mathrm{\,\% }/\left(\text{Kariespr"avalenz in }\mathrm{\% }\right)\times \,\text{mittlere DMFT}$ [5]

**Table 5:** 

Bundesland/Region	*N*	DMFT	IT	DT	MT	FT	SiC	SaC	Sanierungsgrad in %	Kinder mit DT in %	DMFT = 0 in %
Baden-Württemberg	1534	0,38	1,39	0,12	0,01	0,25	1,14	2,14	69,6	6,0	82,3
Bayern	1237	0,62	0,72	0,28	0,01	0,33	1,85	2,18	55,2	13,9	71,7
Berlin	6451	0,74	0,53	0,22	0,03	0,49	2,20	2,19	70,2	13,3	66,4
Brandenburg	6919	0,48	0,20	0,12	0,02	0,34	1,45	2,09	76,1	6,6	76,9
Bremen	1314	0,65	0,58	0,23	0,02	0,40	1,95	1,94	64,8	14,5	66,3
Hamburg	3305	0,39	0,61	0,11	0,01	0,27	1,16	2,01	72,6	6,2	80,7
Hessen	1934	0,38	0,38	0,09	0,02	0,27	1,13	2,06	76,4	5,2	81,6
Mecklenburg-Vorpommern	1864	0,46	0,31	0,07	0,02	0,38	1,38	1,89	85,6	5,2	75,8
Niedersachsen	1479	0,44	0,35	0,12	0,03	0,29	1,31	2,01	71,9	7,6	78,3
Nordrhein	3296	0,38	0,23	0,09	0,02	0,28	1,14	2,03	78,7	5,2	81,3
Rheinland-Pfalz	2809	0,24	0,45	0,08	0,01	0,15	0,71	1,78	65,5	5,6	86,6
Saarland	1795	0,27	0,11	0,08	0,02	0,17	0,81	1,91	69,8	5,4	85,9
Sachsen	2254	0,44	0,17	0,11	0,01	0,33	1,32	2,11	76,0	6,1	79,1
Sachsen-Anhalt	6391	0,52	0,14	0,13	0,02	0,36	1,55	2,21	75,2	7,0	76,7
Schleswig-Holstein	2777	0,33	0,19	0,06	0,01	0,26	0,99	2,07	81,8	3,7	84,0
Thüringen	7539	0,44	0,24	0,12	0,02	0,30	1,31	2,10	72,7	7,0	79,2
Westfalen-Lippe	2114	0,40	0,23	0,11	0,01	0,27	1,19	2,06	70,2	6,7	80,7
Deutschland^a^	*55.002*	0,44	0,52	0,14	0,02	0,29	1,33	2,07	70,3	7,7	78,8

^a^DMFT-Wert für Deutschland nach Bevölkerung gewichtet

*DMFT „*decayed, missing, filled permanent teeth“: Index für die Karieserfahrung im bleibenden Gebiss

*IT* initialkariöser Zahn, *DT* defektkariöser Zahn, *MT* extrahierter Zahn, *FT* gefüllter Zahn

*Sanierungsgrad*: Anteil sanierter Zähne am DMFT

*DMFT* *=* *0:* Anteil gesunder Gebisse in %

*Kinder mit DT*: prozentualer Anteil der Kinder mit unbehandelten defektkariösen Zähnen

*SaC*_*DMFT*_ Specific Affected Caries Index: mittlerer DMFT in der Gruppe mit DMFT > 0 [5]

*SiC*_*DMFT*_ Significant Caries Index: mittlerer DMFT-Wert des einen Drittels der Kinder mit den höchsten Karieswerten [4]

Die von Karies betroffenen Kinder haben im Schnitt (SaC [5]) 2,1 DMFT und dieser Wert liegt somit deutlich über dem Mittelwert von 0,5 DMFT bei allen 12-Jährigen. Zudem waren etwa 30 % der kariösen bleibenden Zähne bei den 12-Jährigen nicht saniert [2]. Die mittleren Karieswerte sind bei Hauptschülern oder Förderschülern deutlich höher als bei Gymnasialschülern (je nach Bundesland ca. 3–5-fach; [2]).

### Zusammenfassung der aktuellen Mundgesundheit bei Kindern in Deutschland

Mit sinkender Kariesprävalenz in Deutschland ist eine zunehmende Polarisation der Karies zu verzeichnen. So tritt Karies an Milchzähnen (Tab. 6) schon früh auf (ca. 10–17 % der 3‑Jährigen bei sehr niedrigem Sanierungsgrad) und ist insgesamt noch weitverbreitet (ca. 40–60 % der 6‑ bis 7‑Jährigen; [2]). Somit lässt sich hier kaum von einem ausschließlich sozialen Randphänomen sprechen. Auch wenn sich die dmft-Werte bei den 6‑ bis 7‑Jährigen seit Beginn der Erfassung 1994 deutlich (um ca. 40 %) reduziert haben, sind jedoch in den letzten 10 Jahren kaum Verbesserungen der Mundgesundheit im Milchgebiss zu verzeichnen [2, 13]. Dies gilt ebenfalls für die erstmalig im Rahmen der DAJ-Studie erfasste Altersgruppe der 3‑Jährigen, deren Prävalenzdaten vergleichbar mit denen früherer regionaler Studien zur ECC sind [13, 18]. Die Karieswerte bei 12-Jährigen in Deutschland sind dagegen extrem gesunken: Im Zeitraum 1994 bis 2016 sank der mittlere DMFT von 2,4 um ca. 80 % auf 0,4 bei knapp 80 % kariesfreien Gebissen (Tab. 4**und** Tab. 6). Diese Beobachtungen decken sich für die 12-Jährigen mit den Daten aus den *Deutschen Mundgesundheitsstudien* [6] und somit liegt Deutschland zusammen mit Dänemark international an der Spitze in dieser Altersgruppe [19].

**Table 6:** 

DAJ-Studie 2016	3‑Jährige	6- bis 7‑Jährige	12-Jährige
Anzahl untersuchter Kinder (*n*)	95.127	151.555	55.002
Mittlere Karieserfahrung in Deutschland	0,48 dmft	1,73 dmft	0,44 DMFT
Spanne mittlerer Karieserfahrung in den verschiedenen Bundesländern	0,4–0,6 dmft	1,4–2,3 dmft	0,24–0,74 DMFT
Kariesprävalenz (Anteil der Kinder mit dmft > 0 bzw. DMFT > 0)	13,7 %	43,6 %	21,2 %
Anteil unsanierter kariöser Zähne (Anteil dt bzw. DT an dmft bzw. DMFT)	73,9 %	42,5 %	29,7 %
Anteil der Kinder mit Sanierungsbedarf (Kinder dt > 0 bzw. DT > 0)	11,4 %	26,9 %	7,7 %
SaC: mittlere Karieserfahrung bei Kindern mit dmft > 0 oder DMFT > 0	3,6 dmft	3,97 dmft	2,07 DMFT

*dmft* „decayed, missing and filled teeth“: Index zur Karieserfahrung im Milchgebiss

*DMFT* „decayed, missing, filled permanent teeth“: Index für die Karieserfahrung im bleibenden Gebiss

## Diskussion

### Vergleichbarkeit der DMFT-Werte aus den verschiedenen Studien

Interessanterweise liegen die Werte für die Kariesprävalenz und die mittlere Karieserfahrung in der Gruppe der 12-Jährigen in 2 unabhängigen Studien mit verschiedener methodischer Herangehensweise (DAJ-Studie [2] und DMS V [6]), deren Untersuchungszeitpunkt nur knapp 2 Jahre auseinanderliegen, sehr nah beieinander (0,44 vs. 0,5 DMFT; 21 % vs. 19 % Kariesprävalenz [2, 6]). Die Karieswerte waren bereits bei der DMS IV (2005) und der vorangegangenen DAJ-Studie (2009) sehr ähnlich (0,72 vs. 0,7 DMFT [6, 22]). Für das Milchgebiss existieren keine anderen nationalen Daten neben der DAJ-Studie. Dennoch liegen die Karieswerte sowohl für das Milchgebiss als auch für die bleibenden Zähne (Tab. 1, 3**und** Tab. 5) in den einzelnen Bundesländern ebenfalls relativ nah beieinander [14] und der Trend ist vergleichbar [13, 21], was neben den validen Werten bei 12-Jährigen ein weiterer Hinweis auf eine robuste Darstellung der Karieserfahrung ist.

Nach einer einfachen Adjustierung des DMFT (Anpassung um 15 %) in der DAJ-Studie wg. der Beschränkung auf Klassenstufe 6 [23] liegt der korrigierte DMFT-Wert bei 0,5, was genau dem Wert in der DMS V entspricht [6]. Der Sanierungsgrad der Zähne war ebenfalls ähnlich (DAJ: 70,5 % vs. IDZ: 74,6 % [2, 6]). Es ist trotzdem kritisch anzumerken, dass aufgrund der schiefen Verteilung von Karies der mittlere DMFT eigentlich nicht (mehr) der korrekte Marker zur Darstellung der Erkrankungslast ist. Nichtsdestoweniger stellt der mittlere DMFT als historisch gewachsener und verwendeter Index [10], wie beschrieben, die Karieserfahrung valide, zuverlässig und einfach verständlich dar, sodass Limitationen [24], die zudem nur teilweise bei der Erfassung von Karies bei Kindern zum Tragen kommen, vor allem im Hinblick auf die Vorteile dieses Index hingenommen werden können.

### Änderungen in den Rahmenbedingungen

Über den Zeitraum von mehreren Jahrzehnten verändern sich selbstverständlich die Rahmenbedingungen im politischen und gesellschaftlichen Umfeld sowie im Gesundheitssystem. Einige Änderungen im zahnärztlichen Setting können einen wesentlichen Einfluss auf die Mundgesundheit ausgeübt haben. So stellt eine Präventionssäule die halbjährliche Individualprophylaxe (IP) dar, die im Jahr 1989 für 12- bis 17-Jährige in den Leistungskatalog der gesetzlichen Krankenversicherung (GKV) integriert wurde. 1993 wurde diese Leistung für alle Kinder ab 6 Jahren zugänglich und um die präventive Fissurenversiegelung an Molaren (IP5) erweitert. Die IP-Leistungen sind wahrscheinlich ein wesentlicher Faktor für die Verbesserung der DMFT-Werte bei 12-Jährigen. 2004 wurden dann auch erstmalig für Kinder ab zweieinhalb Jahren kariespräventive Leistungen in den GKV-Katalog aufgenommen, allerdings in erheblich geringerem Umfang als im IP-Programm [25].

Bei den jüngeren Kindern bestand – wie die DAJ-Studie zeigte – ein polarisierter Kariesbefall im Milchgebiss, welcher ein deutliches Problem darstellt [26]. Hierbei wird es insbesondere problematisch, wenn kariöse Zähne eine Pulpabeteiligung, also Schmerzsymptomatik und z. B. dentogene Fisteln bzw. Abszesse, aufweisen. Aufgrund des geringen Alters, eingeschränkter Kooperationsfähigkeit und deutlicher Erkrankungslast (Polarisation) ist bei vielen dieser von Karies betroffenen Kleinkinder eine umfangreiche Zahnbehandlung nur unter Narkose durchzuführen [27]. Dies ist auf individueller Ebene und gesellschaftlich sicherlich nicht primär erwünscht, denn es führt neben den Narkoserisiken [28] auch zu deutlichen Kosten für das Gesundheitssystem [29].

### Implementierung neuer Präventionsmaßnahmen

Mitte 2019 wurden daher die kariespräventiven Leistungen für das Milchgebiss durch neue zahnärztliche Frühuntersuchungen bereits ab 6 Monaten (FU1a–c) sowie die risikounabhängige Fluoridlackapplikation (FLA) und das praktische Putztraining mit der Betreuungsperson (FUPr) weiter ausgebaut. In der Zukunft wird sich zeigen, inwieweit Karies bei Kleinkindern vermieden werden kann. Denn es muss auch immer berücksichtigt werden, dass die IP im Vergleich zur Gruppen- und Kollektivprävention kostenintensiver und ihr Erfolg abhängig vom Erscheinen der Patienten in der Zahnarztpraxis ist. Dies kann gerade in Risikogruppen problematisch sein [25, 30, 31]. Ein zentraler Baustein für eine optimierte Kariesprävention im Milchgebiss könnte dabei also die Zahnpasta spielen: So enthielt Kinderzahnpasta in Deutschland lange maximal 500 ppm und somit nur ein Drittel des Fluorids von Erwachsenenzahnpasta. Neueste Empfehlungen der deutschen zahnmedizinischen Fachgesellschaften [32] sehen vor, dass bei Kindern ab dem Durchbruch des 1. Milchzahnes bis zum 2. Geburtstag mit einer reiskorngroßen Zahnpastamenge mit 1000 ppm Fluorid geputzt werden sollte. Alternativ dazu kann auch empfohlen werden, zweimal täglich mit einer erbsengroßen Zahnpastamenge mit 500 ppm Fluorid zu putzen. Vom 2. bis 6. Geburtstag sollten die Zähne dann zweimal täglich mit einer erbsengroßen Zahnpastamenge mit 1000 ppm Fluoridgehalt geputzt werden [32].

Aufgrund der klaren Dosis-Wirkung-Beziehung [33] ist es sehr wahrscheinlich, dass ein Teil der Milchgebisskaries bei Erhöhung des Fluoridgehalts in der Kinderzahnpasta reduziert werden kann. Insbesondere bei (Klein‑)Kindern mit erhöhter Kariesaktivität oder erhöhtem Kariesrisiko wäre die Nutzung einer Kinderzahnpasta mit 1000 ppm Fluorid oder gar einer Juniorzahnpasta (1250–1450 ppm) sinnvoll.

In letzter Zeit haben verschiedene Akteure intensiv daran gearbeitet, die bis dato bestehende Präventionslücke bei Kleinkindern zu verkleinern. Neben der Empfehlung eines höheren Fluoridgehalts in der Zahnpasta, also eine Angleichung deutscher Empfehlungen an europäische Standards [34], wurden in der Zahnarztpraxis abrechnungsfähige kariespräventive Maßnahmen im Kleinkindalter bis zweieinhalb Jahre eingeführt. Die Neufassung der FU-Richtlinie beinhaltet neue Frühuntersuchungen, praktisches Training zur Mundhygiene und Fluoridierungsmaßnahmen [35]. Dies war angesichts der hohen Raten von ECC [2] und damit assoziierten Zahnsanierungen in Narkose bei schweren Fällen für ein hochentwickeltes Land überfällig. Zudem sollen Kinderärzte mithilfe der seit 2016 verfügbaren überarbeiteten gelben Kinderuntersuchungshefte während der verpflichtenden U‑Untersuchungen (U5–U7) auf die zahnärztlichen Frühuntersuchungen bei Kleinkindern verweisen und somit die ECC-Prävention fördern [36]. Ein Pilotprojekt in Jena konnte die Wirksamkeit früh ansetzender Prävention bereits zeigen [37].

Oben genannte Maßnahmen stehen im Einklang mit der „International Association for Paediatric Dentistry (IAPD) Bangkok Declaration“ [38] zur ECC. So sollten zur Vermeidung von ECC Eltern, Zahnärzte, Ärzte und viele andere Beteiligte bezüglich ECC sensibilisiert werden, eine vorbeugende Beratung möglichst innerhalb des ersten Lebensjahres des Kindes durch einen Angehörigen eines Gesundheitsberufs erfolgen oder idealerweise ein Verweis an einen Zahnarzt für eine umfassende Betreuung stattfinden. Schlüssel zur Vermeidung von ECC sind „Zweimal täglich Zähne putzen mit fluoridierter Zahnpasta (mindestens 1000 ppm)“ und eine limitierte Zufuhr zuckerhaltiger Getränke und Speisen [38].

### Rolle der Fluoride in der Kariesprävention

Weiterhin geben die evidenzbasierten Empfehlungen eines systematischen Reviews eine gute Übersicht, welche Maßnahmen für das Milchgebiss im Allgemeinen und bei erhöhtem Kariesrisiko sowohl in der Praxis als auch häuslich eingesetzt werden sollten [39]. Diese Empfehlungen weisen Fluorid in diversen Applikationsformen eine zentrale Bedeutung zu. Hier sollte der primäre Fokus in der Kariesprävention liegen. Andere Maßnahmen sind eher additiv. Eine hohe Fluoridierungsquote wäre auch für eine zeitgemäße und wirksame Gruppenprophylaxe essenziell [40], wie erfolgreiche Pilotprogramme in Greifswald oder Osnabrück-Land belegen [41, 42]. Die sich zurzeit in Überarbeitung befindende deutsche Leitlinie zu den Fluoridierungsmaßnahmen zur Kariesprophylaxe wird voraussichtlich auch Annäherungen an die internationalen Empfehlungen mit Verschiebungen zur Fluoridzahnpasta enthalten, da bei Fluoriden die lokale Applikation vorrangig genutzt werden sollte [40].

### Aktionsplan und Ausblick

Ein stringenter evidenzbasierter Handlungsplan „Prävention im Milchgebiss“ wäre daher für Deutschland sinnvoll. Dieser umfasst den frühen Verweis vom Kinderarzt zum Zahnarzt, die Erhöhung des Fluoridgehalts auf 1000 ppm in der Kinderzahnpasta, die Nutzung der neuen Früherkennungsuntersuchungen (FUs) und die Fluoridlackapplikation bei Kindern. Dabei sollten alle Möglichkeiten der Kollektiv‑, Gruppen- und Individualprophylaxe genutzt werden [25]. Wie bereits beschrieben sind in den letzten Jahren einige wichtige Strategien entwickelt und Präventionsmaßnahmen insbesondere für das Milchgebiss implementiert worden [32, 35, 36].

Der positive Mundgesundheitstrend in der Gruppe der 12-Jährigen wird sich wohl noch weiter fortsetzen, auch wenn die Kurve abzuflachen scheint [20, 21], da für eine wirksame Prävention in der verbliebenen Kariesrisikogruppe bei 12-Jährigen andere Strategien notwendig sind. Das Optimierungspotenzial der Mundgesundheit bei 12-Jährigen in Deutschland ist aufgrund der niedrigen Karieswerte [2, 6] deutlich kleiner und eine weitere Verbesserung wohl fast nur über kariesrisikospezifische Präventionsmaßnahmen in Risikoeinrichtungen/Schulen möglich, wie z. B. regelmäßiges Zähneputzen und zusätzlich hochfrequente Applikationen höher konzentrierter Fluoridpräparate (z. B. durch Einbürstung von Fluoridgelee; [43]).

Welchen genauen Einfluss die neu eingeführten oben genannten Maßnahmen, wie beispielsweise der Verweis vom Kinderarzt zum Zahnarzt, der erhöhte Fluoridgehalt (1000 ppm) in der Kinderzahnpasta, die neuen Früherkennungsuntersuchungen und die Einführung der Fluoridlackapplikation bei Kindern unter 6 Jahren, auf die Kariesprävalenz haben werden, ist nicht genau vorhersagbar. Jedoch wird wahrscheinlich bereits bei den nächsten Untersuchungen im Rahmen der *Epidemiologischen Begleituntersuchungen zur Gruppenprophylaxe* eine (leichte) Verbesserung zu verzeichnen sein.

## Fazit

- Die kariesepidemiologische Literatur zeigt, dass Karies im Milchgebiss immer noch hochprävalent ist, bei Jugendlichen dagegen nur noch relativ kleine Kariesrisikogruppen existieren. Durch früh ansetzende Kariesprävention, Empfehlungen für einen höheren Fluoridgehalt (1000 ppm) in der Kinderzahnpasta, regelmäßige Verweise der Kleinkinder vom Kinderarzt zum Zahnarzt für adäquate zahnärztliche Frühuntersuchungen schon ab dem 6. Lebensmonat und verbesserte Leistungen in der zahnärztlichen IP scheinen die Ziele, ECC sowie Milchzahnkaries in allen Altersgruppen zu reduzieren, näher zu rücken. Für eine weitere Verbesserung der Zahngesundheit in der permanenten Dentition sollte der Fluorideinsatz insbesondere in Schulen mit höherem Anteil an Kariesrisikogruppen über das tägliche Zähneputzen und weitere Fluoridimpulse mit höher konzentrierten Fluoridpräparaten implementiert werden. Ein regelmäßiges Monitoring der Mundgesundheit ist lokal, aber auch deutschlandweit weiterhin nötig.
- Diese gemeinsam erzielte, aktuell schon recht gute Mundgesundheit bei Kindern und die bislang neu implementierten strukturellen Maßnahmen stellen ein gutes Beispiel für die Innovationsfähigkeit der Zahnmedizin dar, bei der weitere Verbesserungen, insbesondere für das Milchgebiss, realistisch erscheinen und in den nächsten Jahren zu erwarten sind.
